
JOURNAL INFORMATION
==============================
NLM Title Abbreviation: ScientificWorldJournal
Journal ID: ScientificWorldJournal
NLM Journal ID: 101131163
Journal ID: TSWJ

The Scientific World Journal

ISSN: 2356-6140
EISSN: 1537-744X
Publisher: Wiley

ARTICLE INFORMATION
==============================
PMCID: PMC5917279
PMID: 16565772
DOI: 10.1100/tsw.2006.66
Article ID: 985940
Article version: 1
Subjects: Directions in Science

New Perspectives in Chondrogenic Differentiation of Stem Cells for Cartilage Repair

Toh Wei Seong 1
Yang Zheng 1 2
Heng Boon Chin 1
Cao Tong 1 *
1 Stem Cell Laboratory, Faculty of Dentistry, National University of Singapore, Singapore 117510,
2 Department of Orthopaedic Surgery, Yong Loo Lin School of Medicine, NUS Tissue Engineering Program, National University of Singapore, 27 Medical Dr, Singapore 117510,
*Tong Cao: dencaot@nus.edu.sgAcademic Editor: Martin Goette

Publication date: 2006
Electronic publication date: 2006 Mar 20
Volume: 6
First page: 361
Last page: 364
Received 2006 Feb 17; Revised 2006 Feb 26; Accepted 2006 Mar 6
Copyright: Copyright © 2006 Wei Seong Toh et al.
Copyright year: 2006
License: This is an open access article distributed under the Creative Commons Attribution License, which permits unrestricted use, distribution, and reproduction in any medium, provided the original work is properly cited.
License URL: https://creativecommons.org/licenses/by/3.0/

Keywords: TGFβ1, BMP2, chondrogenic, embryonic, human, stem cells

==============================
Directions in Science
*TheScientificWorldJOURNAL* (2006) 6, 361–364
ISSN 1537-744X; DOI 10.1100/tsw.2006.66

TheScientificWorldJOURNAL
www.thescientificworld.com

### New Perspectives in Chondrogenic Differentiation of Stem Cells for Cartilage Repair

Wei Seong Toh[1], Zheng Yang[1,2], Boon Chin Heng[1], and Tong Cao[1,*]

*[1]Stem Cell Laboratory, Faculty of Dentistry, National University of Singapore, 27 Medical Dr, Singapore 117510; [2]Department of Orthopaedic Surgery, Yong Loo Lin School of Medicine, NUS Tissue Engineering Program, National University of Singapore, 27 Medical Dr, Singapore 117510*

E-mail: g0300293@nus.edu.sg; dosyz@nus.edu.sg; denhenga@nus.edu.sg; dencaot@nus.edu.sg

*Received February 17, 2006; Revised February 26, 2006; Accepted March 6, 2006; Published March 20, 2006*

**KEYWORDS**: TGFβ1, BMP2, chondrogenic, embryonic, human, stem cells

Traumatic injury and age-related degenerative diseases associated with cartilage are major health problems worldwide[1,2] due to the limited self-regenerative capacity of cartilaginous tissue. Cell-based therapy and tissue engineering have been widely proposed as means of cartilage reconstruction[3]. These usually involve the *ex vivo* expansion of autologous chondrocytes[4] or bone marrow–derived mesenchymal stem cells (BM-MSC)[5], followed by subsequent reimplantation at the site of cartilage injury, either as a cell suspension or in association with collagen gels, hydrogels, or synthetic biodegradable scaffolds[6].

Human mesenchymal stem cells (hMSCs) that have the ability to self-renew and give rise to various cell types of the mesenchymal lineage, including adipocytes, chondrocytes, and osteoblasts, are an attractive cell source for tissue engineering and regenerative medicine[5]. However, an optimized and defined culture milieu for directing these cells into the chondrogenic lineage is still lacking[7]. Recently, BM-MSCs were used for autologous repair of cartilage defects in the osteoarthritic knee[8]. However, clinical evaluation of the repaired tissue revealed formation of hyaline cartilage in only some areas of the regenerate, and in most cases, the penetration of subchondral bone and subsequent transplantation or recruitment of MSCs resulted in the formation of inadequate fibrocartilaginous repair tissue with the accumulation of type I collagen[8,9]. Moreover, hMSC-derived chondrogenic cells have the ability to further progress to the hypertrophic state, which undergoes cartilaginous erosion and vascularization, resembling the endochondral ossification process, which is recaptitulated during the development of osteoarthritis[10]. This phenomenon suggests that hMSCs may, in fact, be programmed for the formation of transient cartilage destined to form bone, and unless appropriately controlled, may not be a ready precursor of permanent articular cartilage. In the developing embryonic limb bud, articular cartilage cells are derived from undifferentiated mesenchymal condensation cells at very early stage of joint formation, as a result of formation of the inter-zone[11,12]. However, once synovial joint formation is completed, articular cartilage cells are no longer derived from mesenchymal condensation cells[13,14], while chondrocytes derived from the remaining mesenchymal condensations will eventually undergo hypertrophy and endochondral ossification[14,15]. This developmental pathway also suggests that hMSCs in adult bone marrow may not be able to differentiate into articular cartilage without progression

*Corresponding author
©2006 with author.
Published by TheScientificWorld, Ltd.; www.thescientificworld.com to full maturation. Hence, understanding the factors, signals, or even the environment that induce MSCs towards specific chondrogenic lineage development and subsequent maintenance of the cartilage phenotype is necessary prior to clinical application. Recent work done by our group[16], together with work by other groups[17,18], suggest possible combined effects of TGFβ1 and BMP2 in chondrogenic differentiation of MSCs that result in hyaline-like cartilage formation, with minimal progression to hypertrophy.

An alternative cell source for cartilage repair, other than primary explanted chondrocytes and BM-MSC, are human embryonic stem cells (hESCs) and stem cell lines derived from the inner cell mass of blastocyst-stage embryos[19]. hESCs hold great promise to become an unprecedented system for gaining insight into the molecular and cellular mechanisms that dictate lineage specification during early stages of human development. In addition, the most obvious advantages of hESCs are their pluripotency and immortality, which could potentially provide an unlimited stable supply of chondroprogenitor cells and differentiated chondrocytes for transplantation therapy.

Our group's prevailing research interests involve utilizing hESCs as a model system of analysis to understand the microenvironment, growth factors, and signaling pathways that direct and control early osteochondrogenic differentiation and commitment. Current studies in our laboratory have shown that BMP2 is able to induce chondrogenic differentiation and maturation/hypertrophy of human embryoid body (hEB)-derived cells in both 2-D EB monolayer and 3-D high-density micromass culture systems, of which a distinct temporal pattern of chondrogenic activation and hypertrophic development was observed. BMP2 induced an earlier and more robust chondrogenic activation and hypertophic development in high-density micromass system, when compared to the monolayer system. This clearly demonstrated the importance of cell-to-cell contact, which makes the high-density micromass system a more appropriate system to study chondrogenesis (Toh, W.S. et al. submitted for publication). Notably, further analysis suggested that BMP2 exerts a bipotent effect in inducing both chondrogenic and osteogenic differentiation, while TGFβ1 played a stirring effect in driving chondrogenesis predominately, with inhibition of osteogenesis. Our results also indicate that TGFβ1 alone could be sufficient to promote chondrogenesis of hEB-derived cells, from chondrogenic activation to maturation to become hypertropic chondrocytes. Both factors revealed little synergistic cooperation in enhancing chondrogenic differentiation, although BMP2 promotes hypertrophic development in synergy with TGFβ1, a phenomenon that was not observed in BM-MSCs[17]. This finding is consistent with previous studies on human embryonic germ cells[20] and mouse embryonic stem cells[21] which showed that a combination of BMP and TGFβ does not have a cooperative effect in enhancing chondrogenic differentiation.On the other hand, it was well-documented in several studies[22,23] that different isoforms of BMPs, such as BMP2 and BMP6, interact in a synergistic fashion with TGFβ in the enhancement of chondrogenic differentiation of hMSCs. These findings suggested the disparate effects of growth factors on stem cells of embryonic and adult origin during the course of development. It is also important to note in this context that ESCs represent a pluripotent cell source with the ability of differentiating into a far wider range of lineages compared to adult stem cells such as MSCs, which are committed primarily to mesenchymal cell lineages. Furthermore, one has to consider that EBs contain a variety of differentiated cell types and in a culture system whereby hEB-derived cells were induced into the chondrogenic lineage by specific growth factors, the interaction among different lineages and how each may affect another during the course of development remains to be elucidated.

Eventual application of hESCs in cell-based cartilage regeneration will depend largely on our ability to differentiate hESCs specifically into a specific lineage of interest, followed by purification of the desired cell population. Molecular and genetic manipulation (gain- and loss-of-function) on signaling transduction pathways implicated in chondrogenesis will have to be explored. Purification of lineage-specific subpopulations of interest from the bulk population of differentiating hESCs and hEBs represents another critical step for any potential gene and cell therapy strategies. This could be achieved by the introduction of reporter/selector genes, which are rigidly expressed under the control of lineage-specific promoters to track the lineage commitment of hESCs[24]. Looking to the future, generation of chondrogenic-specific collagen II-promoter-driven green fluorescence protein expression system in hESCs may be a promising and feasible strategy for identification and selection of putative chondroprogenitors and chondrocytes at different stages of differentiation for further characterization and eventual clinical application.

### REFERENCES

1. Buckwalter, J.A. (2002) Articular cartilage injuries. *Clin. Orthop*. 21–37.
2. Martin, J.A. and Buckwalter, J.A. (2002) Aging, articular cartilage chondrocyte senescence and osteoarthritis. *Biogerontology* **3**, 257–264.
3. Cancedda, R., Dozin, B., and Giannoni, P. (2003) Tissue engineering and cell therapy of cartilage and bone. *Matrix Biol*. **22**, 81–91.
4. Brittberg, M., Lindahl, A., and Nilsson, A. (1994) Treatment of deep cartilage defects in the knee with autologous chondrocyte transplantation. *N. Engl. J. Med*. **331**, 889–895.
5. Tuan, R.S., Boland, G., and Tuli, R. (2003) Adult mesenchymal stem cells and cell-based tissue engineering. *Arthritis Res. Ther*. **5(1),** 32–45.
6. Frenkel, S.R. and Di Cesare, P.E. (2004) Scaffolds for articular cartilage repair. *Ann. Biomed. Eng.* **32,** 26–34
7. Heng, B.C., Cao, T., and Lee, E.H. (2004) Directing stem cell differentiation into the chondrogenic lineage in vitro. *Stem Cells* **22(7),** 1152–1167.
8. Wakitani, S., Imoto, K., Yamamoto, T., Saito, M., Murata, N., and Yoneda, M. (2002) Human autologous culture expanded bone marrow mesenchymal cell transplantation for repair of cartilage defects in osteoarthritic knees. *Osteoarthritis Cartilage* **10(3),** 199–206.
9. Wakitani, S., Mitsuoka, T., Nakamura, N., Toritsuka, Y., Nakamura, Y., and Horibe, S. (2004) Autologous bone marrow stromal cell transplantation for repair of full-thickness articular cartilage defects in human patellae: two case reports. *Cell Transplant*. **13(5),** 595–600.
10. Wieland, H.A., Michaelis, M., Kirschbaum, B.J., and Rudolphi, K.A. (2005) Osteoarthritis - an untreatable disease? *Nat. Rev. Drug Discov*. **4**, 331–344.
11. Archer, C.W., Dowthwaite, G.P., and Francis-West, P. (2003) Development of synovial joints. *Birth Defects Res. C Embryo Today* **69**, 144–155.
12. Karsenty, G. and Wagner, E.F. (2002) Reaching a genetic and molecular understanding of skeletal development. *Dev. Cell* **2**, 389–406.
13. Murphy, J.M., Heinegard, R., McIntosh, A., Sterchi, D., and Barry, F.P. (1999) Distribution of cartilage molecules in the developing mouse joint. *Matrix Biol*. **18**, 487–497.
14. Hayes, A.J., MacPherson, S., Morrison, H., Dowthwaite, G., and Archer, C.W. (2001) The development of articular cartilage: evidence for an appositional growth mechanism. *Anat. Embryol. (Berl.)* **203**, 469–479.
15. Pacifici, M., Koyama, E., Iwamoto, M., and Gentili, C. (2000) Development of articular cartilage: what do we know about it and how may it occur? *Connect. Tissue Res*. **41**, 175–184.
16. Toh, W.S., Liu, H., Heng, B.C., Rufaihah, A.J., Ye, C.P., and Cao, T. (2005) Combined effects of TGFbeta1 and BMP2 in serum-free chondrogenic differentiation of mesenchymal stem cells induced hyaline-like cartilage formation. *Growth Factors* **23(4),** 313–321.
17. Schmitt, B., Ringe, J., Haupl, T., Notter, M., Manz, R., Burmester, G.R., Sittinger, M., and Kaps, C. (2003) BMP2 initiates chondrogenic lineage development of adult human mesenchymal stem cells in high-density culture. *Differentiation* **71(9–10),** 567–577.
18. Hanada, K., Solchaga, L.A., Caplan, A.I., Hering, T.M., Goldberg, V.M., Yoo, J.U., and Johnstone, B. (2001) BMP-2 induction and TGF-beta 1 modulation of rat periosteal cell chondrogenesis. *J. Cell. Biochem*. **81(2),** 284–294.
19. Thomson, J.A., Itskovitz-Eldor, J., Shapiro, S.S., Waknitz, M.A., Swiergiel, J.J., Marshall, V.S., and Jones, J.M. (1998) Embryonic stem cell lines derived from human blastocysts. *Science* **282,** 1145–1147
20. Kim, M.S., Hwang, N.S., Lee, J., Kim, T.K., Leong, K., Shamblott, M.J., Gearhart, J., and Elisseeff, J. (2005) Musculoskeletal differentiation of cells derived from human embryonic germ cells. *Stem Cells* **23(1),** 113–123.
21. Hwang, N.S., Kim, M.S., Sampattavanich, S., Baek, J.H., Zhang, Z., and Elisseeff, J. (2006) The effects of three dimensional culture and growth factors on the chondrogenic differentiation of murine embryonic stem cells. *Stem Cells* **24(2),** 284–291
22. Sekiya, I., Larson, B.L., Vuoristo, J.T., Reger, R.L., and Prockop, D.J. (2005) Comparison of effect of BMP-2, -4, and -6 on in vitro cartilage formation of human adult stem cells from bone marrow stroma. *Cell Tissue Res*. **320(2),** 269–276.
23. Indrawattana, N., Chen, G., Tadokoro, M., Shann, L.H., Ohgushi, H., Tateishi, T., Tanaka, J., and Bunyaratvej, A. (2004) Growth factor combination for chondrogenic induction from human mesenchymal stem cell. *Biochem. Biophys. Res. Commun*. **320(3),** 914–919.
24. Menendez, P., Wang, L., and Bhatia, M. (2005) Genetic manipulation of human embryonic stem cells: a system to study early human development and potential therapeutic applications. *Curr. Gene Ther*. **5(4),** 375–385.

**This article should be cited as follows:**

Toh, W.S., Yang, Z., Heng, B.C., and Cao, T. (2006) New perspectives in chondrogenic differentiation of stem cells for cartilage repair. *TheScientificWorldJOURNAL* **6**, 361–364. DOI 10.1100/tsw.2006.66.

### BIOSKETCHES

**Wei Seong Toh** graduated with a BSc from The University of Melbourne, Australia in 2003. He holds a NUS research scholarship and is currently undertaking a postgraduate research on the stimulations and modulations of chondrogenesis of embryonic and adult stem cells in the Stem Cell Laboratory, Faculty of Dentistry, National University of Singapore, and will be awarded his MSc from National University of Singapore in July 2006. He will continue to pursue his research interest in establishing chondrogenic hESC clones for selection of chondrogenic cells and studies of hESC chondrogenic differentiation and maturation by undertaking a PhD in National University of Singapore.

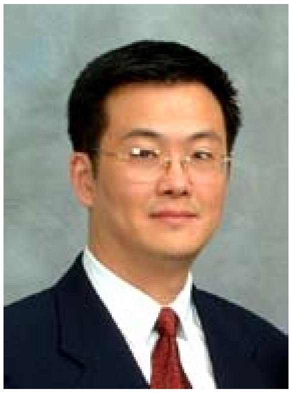

**Dr. Tong Cao** is an Assistant Professor in the Faculty of Dentistry, National University of Singapore. He obtained his DDS degree at Zhejiang University in Hangzhou, China, and his PhD degree and Adv. Cert. of Oral and Maxillofacial Surgery at the Showa University in Tokyo, Japan. His primary research interests include the somatic differentiation of human embryonic stem cells, the stem cell–based tissue regeneration, the immunointeraction of stem cells in allogenic immune response, the stem cell models for toxicity screening/testing, and the bio-scaffold system for regenerative medicine. Dr. Tong Cao is the Principle Investigator of the Stem Cell Group, Faculty of Dentistry, National University of Singapore.
